# Efficacy and safety of low dose alteplase for intravenous thrombolysis in Asian stroke patients: a meta-analysis

**DOI:** 10.1038/s41598-017-16355-9

**Published:** 2017-11-22

**Authors:** Ge Tan, Haijiao Wang, Sihan Chen, Deng Chen, Lina Zhu, Da Xu, Yu Zhang, Ling Liu

**Affiliations:** Department of Neurology, West China Hospital, Sichuan University, No. 37, Guo Xue Xiang, Chengdu, 610041 Sichuan Province China

## Abstract

Whether low dose alteplase is comparable to standard dose in efficacy and safety for intravenous thrombolysis (IVT) in Asian stroke patients remains unverified. PubMed, EMBASE, and Cochrane Library Database from the beginning to June 30, 2017 were searched. IVT efficacy was measured by favorable outcome (modified Rankin Scale scores of 0–1) at 3 months, and safety measured by mortality within 3 months and symptomatic intracerebral hemorrhage (SICH). Pooled estimates were conducted using fixed- or random-effects model depending on heterogeneity. For SICH, studies were pooled separately according to different definitions. Twelve studies involving 7,905 participants were included. No association was found between alteplase dose and favorable outcome (OR = 0.94, 95% CI 0.78–1.14, *P* = 0.5; heterogeneity: *P*_*hetero*_ = 0.01, I^2^ = 57.3%) and mortality (OR = 0.87, 95% CI 0.74–1.02, *P* = 0.08; *P*_*hetero*_ = 0.83, I^2^ = 0) using random- and fixed-effects models, respectively. Low dose alteplase was associated with lower SICH as defined by the National Institute of Neurological Disorders and Stroke study (OR = 0.79, 95% CI 0.64–0.99, *P* = 0.04; *P*_*hetero*_ = 0.57, I^2^ = 0) using fixed-effects model. Subgroup and sensitivity analysis could change the results significantly. Current limited evidence was insufficient to support the speculation that low dose alteplase was comparable to standard dose in thrombolytic efficacy and safety in Asian stroke patients.

## Introduction

Intravenous thrombolysis (IVT) with alteplase (recombinant tissue plasminogen activator, rt-PA) has been the standard of care for acute ischemic stroke (AIS) since it was approved by the Food and Drug Administration (FDA) in 1996^1,2^. The current recommended dose of rt-PA in the United States and Europe is 0.9 mg/kg with a maximum dose of 90 mg^1^. This recommendation was based on two small dose-escalation trials done in Western countries in the early 1990s^3,4^. However, rt-PA is not without potential adverse consequences, one of the most serious being an increased risk of intracerebral hemorrhage^1,5,6^. And compared to patients from Western countries, Asian patients may have higher risk of intracerebral hemorrhage after thrombolysis^7,8^. Based on the results from the Safe Implementation of Thrombolysis in Stroke-Monitoring study (SITS-MOST) in 2007 and the Safe Implementation of Thrombolysis in Stroke-Non-European Union World (SITS-NEW) study in 2014, the incidence of SICH ranged from 1.66% to 7.27% in European population and from 1.87% to 8.70% in Asian population according to different criteria^9,10^. This increased risk may be attributed to a difference in blood coagulation-fibrinolysis factors, intracranial atherosclerotic diseases, or other physical conditions^8,11^.

Following the Japan Alteplase Clinical Trial (J-ACT), a series of single-arm trials were conducted in Japan and demonstrated that AIS patients receiving rt-PA at a dose of 0.6 mg/kg could obtain comparable efficacy and safety to historical controls given 0.9 mg/kg rt-PA^12–16^. Consequently, the Japanese drug safety authority has approved rt-PA to be administered at a dose of 0.6 mg/kg. It has also become increasingly accepted in other Asian countries to administer a lower dose of rt-PA to AIS patients. However, although many cohort studies directly comparing the efficacy and safety of rt-PA at different doses have been conducted since 2006, results were inconsistent among studies^17–31^. A meta-analysis published in 2015 demonstrated that low dose rt-PA (<0.85 mg/kg) was comparable to standard dose (0.85 mg/kg) in terms of efficacy and safety for IVT^32^. But since then, a randomized study conducted by Anderson *et al*. using data from international multicenter Enhanced Control of Hypertension and Thrombolysis Stroke (ENCHANTED) study, which is the largest randomized trial published to date involving predominantly Asian patients, demonstrated that IVT at a dose of 0.6 mg/kg caused significantly lower risk of symptomatic intracerebral hemorrhages (SICH), but also showed a downward trend in disability-free survival at 3 months when compared to IVT at a dose of 0.9 mg/kg^19^. Thus, whether low dose rt-PA has a better or at least comparable efficacy and safety profile to the standard dose remains unclear.

Almost all studies on this topic were conducted in Asia. Although many studies from Western countries have concentrated on IVT outcomes in patients who are overweight or obesity, or explored the impact of inaccurate weight assessment on IVT outcome^33–44^, there is a lack of studies that perform a head-to-head comparison of the efficacy and safety of different doses of rt-PA. We therefore conducted a meta-analysis to assess the association between rt-PA dose and IVT outcomes for AIS patients, particularly in Asian population.

## Methods

Ethical approval was not necessary for the present study due to no patient involvement. The study was conducted in accordance with the MOOSE (meta-analysis of observational studies in epidemiology protocol) guidelines^45^.

### Literature Search Strategy

A systematic search of the existing literature was performed using PubMed (1976 to June 30, 2017), EMBASE (1982 to June 30, 2017), and the Cochrane Library Database (1987 to June 30, 2017) with no language restriction. Search terms included “stroke”, “ischemic stroke”, “cerebral infarction”, “brain infarction”, “thrombolysis”, “thrombolytic”, “tissue plasminogen activator”, “alteplase”, “rt-PA”, “t-PA”, “dose” and “dosing”. The references from all included studies or relevant reviews were screened to avoid accidental omission.

### Inclusion Criteria

Included studies met the following criteria: (1) type of study: prospective or retrospective study; (2) study population: patients with AIS receiving IVT with rt-PA; (3) comparison: head-to-head comparison in outcomes between different doses; (4) outcome measures: IVT efficacy measured by favorable outcome (modified Rankin Scale [mRS] scores of 0–1) at 3 months, and IVT safety measured by 3-months mortality and SICH after thrombolysis. SICH was defined by criteria from the European Cooperative Acute Stroke Study (ECASS) II^46^, ECASS III^2^, National Institute of Neurological Disorders and Stroke study (NINDS)^1^, and Safe Implementation of Thrombolysis in Stroke-Monitoring study (SITS-MOST)^9^, or by criteria defined by the authors themselves in their researches^22,29,30^.

### Data Extraction and Quality Assessment

A standardized data collection sheet was used to extract data. The following information was collected: publication reference, first author, year of publication, region, number of study centers, design, method of data collection, gender distribution of participants, loss to follow-up, dose of rt-PA, number of cases, age at stroke onset, baseline National Institute of Health Stroke Scale (NIHSS) score, time interval from stroke onset to IVT, and outcome measures (favorable outcome, mortality, and SICH) (Supplementary Table 1). As for SICH, when one study reported the results according to both ECASS II and ECASS III definitions, we would only extract the data of SICH as defined by ECASS II criteria.

Quality of the included studies was assessed using the Newcastle-Ottawa Scales (NOS) items (http://www.ohri.ca/programs/clinical_epidemiology/oxford.asp).

Two investigators (G.T., H.W.) independently selected eligible studies, extracted data, and assessed the quality of articles. Any disagreement between the two investigators was resolved by discussion with the help of a third investigator (L.L.). If there was unavailable data or uncertain information in any of the included studies, the authors would be contacted.

### Group Assignment

IVT with rt-PA at a dose of 0.85 mg/kg was reported to have similar efficacy as that at a dose of 0.95 mg/kg, and SICH seldom occurred with rt-PA doses of ≤0.85 mg/kg^3,4^. We therefore selected 0.85 mg/kg as the cut-off point and divided patients into the low dose (<0.85 mg/kg) or the standard dose group (≥0.85 mg/kg).

### Statistical Analysis

The numbers of patients with the outcomes of interest in the low dose and standard dose groups were extracted. The odds ratio (OR) of low to standard dose with 95% confidential interval (CI) was calculated for each included study. Pooled ORs with 95% CI were calculated from fixed- or random-effects model depending on the heterogeneity among studies. The between-study heterogeneity was assessed by Cochran Q test and I^2^. For Cochran Q test, heterogeneity was considered to be statistically significant when *P*_hetero_ < 0.1. For I^2^, no evidence of heterogeneity was defined as I^2^ of 0, low level of heterogeneity as I^2^ of <25%, moderate level of heterogeneity as I^2^ of 25–50%, and high level of heterogeneity as I^2^ of >50%. A fixed-effects model was applied when *P*_hetero_ > 0.1 or I^2^ < 50%, and a random-effects model was also conducted to evaluate the stability of the results. For SICH, a pooled estimate was conducted by separately synthesizing studies according to different definitions (i.e. ECASS, NINDS, or SITS-MOST).

A subgroup analysis was conducted based on the number of recruited centers, i.e. multicenter (>3 centers) versus non-multicenter. Another subgroup analysis with non-Asian patients excluded were also conducted. Sensitivity analyses, in addition to the switching between a fixed- and random-effects model, were performed as follows: assessing the influence of a single study on the pooled estimate by eliminating one study each time, or assessing the stability of the pooled estimate by removing some of the studies from the meta-analysis according to different exclusion criteria each round.

Potential publication biases were roughly assessed by visual inspection of funnel plots and further identified by Egger’s linear regression test. A *P*-value < 0.05 was considered statistically significant.

All statistical analyses were conducted using the STATA software package (version 14.0, Stata Corporation, College Station, TX, USA).

## Results

### Literature Search and Selection

**Figure 1:**
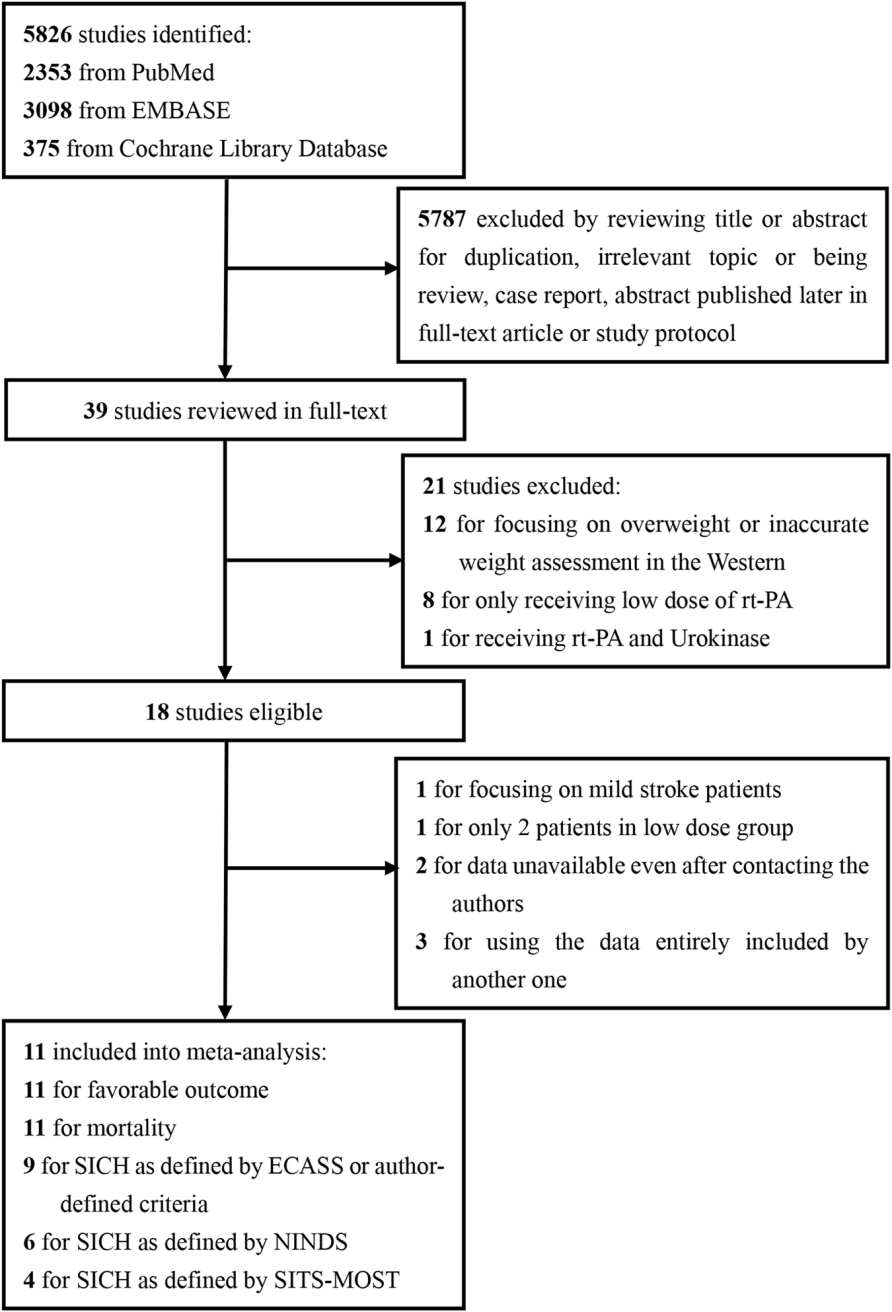
Flow diagram of literature search and selection.

The literature search and selection process are depicted in the flow diagram (Fig. 1). A total of 5,826 citations were initially retrieved. Of them, 5,787 studies were removed by reviewing the title or abstract, leaving 39 studies to be reviewed by the full-text article. Of the 39 studies, 21 were eliminated for not fulfilling the inclusion criteria. Of the remaining 18 studies, 7 were excluded (1 for focusing on patients with only mild stroke^47^, 1 for only including 2 patients who received low dose rt-PA^48^, 2 for having unavailable data even after contacting the author^49,50^, and 3 for using the data that were completely included in another one with a larger sample size^17,19,20,24,28^). Eleven studies were included in the final meta-analysis^18,19,21–27,29,30^.

Among authors contacted for data unavailable or information uncertain, Anderson and his colleague assured us that both Robinson’s study in 2017 and their study in 2016 were conducted using the same data from ENCHANTED study, and participants in Robinson’s study were entirely included in their study^17,19^. They also supplied us the data of favorable outcomes at 3 months for Asian patients. Chao *et al*. replied and acknowledged that data from their 2010 publication was included in whole in another paper published in 2014^24,28^. And the participants in Ho’s study was identified to be the same as that in Chao’s 2010 publication by comparing the data between them^20,28^. Ong *et al*. supplied us data for outcomes of interest and rate of loss to follow-up at 3 months^18^. Abraham *et al*. responded to our data request, but could not supply the missing information on outcomes due to the retrospective design of the study^49^. No reply was obtained from the rest of authors contacted.

### Study Characteristics and Quality

General characteristics of included studies published from 2010 to 2017, are summarized in Supplementary Table 1. Ten studies were conducted in Asia, with 7 from China, and 1 each from Korean, Singapore and Vietnam. The remaining one was performed using an international, multicenter database with 63.2% of the patients being from Asia. Sample size among the studies ranged from 83 to 3,297, with a total of 7,905 participants. The dose of rt-PA across the studies ranged from 0.5 mg/kg to >0.95 mg/kg. The cut-off points of rt-PA dose and groups that participants were divided into were various across included studies. There was a significant between-group difference in gender in 5 studies^22,23,25,27,29^, age in 3 studies^18,24,30^, time interval from stroke onset to IVT in 2 studies^21,24^, and baseline NIHSS score in 1 study^30^. One study had no significant differences in baseline information between the groups being compared^19^.

NOS scores of the included studies are summarized in Supplementary Table 2 and, ranged from 6 to 9 with a mean of 7.7.

### Pooled Estimates for Outcomes

Favorable outcome at 3 months was reported in 11 studies, 7 of which showed a fewer favorable outcome in those receiving the low dose rather than standard dose rt-PA, with one study reaching statistical significance. The remaining 4 studies showed results to the contrary with two study reaching statistical significance. Heterogeneity across the studies was high (*P*_*hetero*_ = 0.01, I^2^ = 57.3%). Using a random-effects model, there was no significant difference in favorable outcome between low dose and standard dose (OR = 0.94, 95% CI 0.78–1.14, *P* = 0.5), as shown in Table 1 and Supplementary Figure 1.

**Table 1:** Overall and subgroup analysis by number of study centers.

Subgroup	Outcomes	NO. of Studies	NO. of Patients	NO. of low dose	NO. of standard dose	*P*_*hetero*_	I^2^	Fixed OR (95% CI)	Random OR (95% CI)
Overall	Favorable outcome	11	7598	1485/3425	1849/4173	0.01	57.3%	0.91 (0.83–1.00)	0.94 (0.78–1.14)
	Mortality	11	7763	303/3534	448/4229	0.83	0	0.87 (0.74–1.02)	0.87 (0.74–1.02)
	SICH by ECASS	9	7424	152/3324	210/4100	0.02	57.9%	0.93 (0.75–1.16)	1.02 (0.66–1.58)
	SICH by NINDS	6	5634	159/2665	206/2969	0.57	0	**0.79 (0.64**–**0.99)**	**0.79 (0.64**–**0.99)**
	SICH by SITS-MOST	4	5446	40/2547	55/2899	0.04	64.0%	0.82 (0.54–1.25)	1.12 (0.49–2.53)
Multicenter* (>3 centers)	Favorable outcome	4	6438	1136/2731	1644/3707	0.61	0	**0.88 (0.80**–**0.98)**	**0.88 (0.80**–**0.98)**
	Mortality	4	6603	263/2840	405/3763	0.69	0	0.87 (0.74–1.03)	0.87 (0.74–1.03)
	SICH by ECASS	4	6711	131/2892	197/3819	0.01	72.9%	0.91 (0.72–1.14)	1.03 (0.64–1.67)
	SICH by NINDS	3	5185	142/2442	195/2743	0.39	0	**0.77 (0.61**–**0.96)**	**0.77 (0.61**–**0.97)**
	SICH by SITS-MOST	3	5185	36/2442	53/2743	0.05	66.6%	0.75 (0.48–1.16)	0.94 (0.40–2.17)
Non-multicenter	Favorable outcome	7	1160	349/694	205/466	0.01	68.0%	1.12 (0.87–1.45)	1.09 (0.67–1.75)
	Mortality	7	1160	40/694	43/466	0.62	0	0.80 (0.49–1.30)	0.84 (0.51–1.40)
	SICH by ECASS	5	713	21/432	12/281	0.09	49.7%	1.22 (0.58–2.52)	1.02 (0.31–3.32)
	SICH by NINDS	3	449	17/223	11/226	0.63	0	1.20 (0.53–2.72)	1.20 (0.52–2.75)
	SICH by SITS-MOST	1	261	4/105	2/156	—	—	3.05 (0.55–16.96)	(0.55–16.96)

ECASS, the European Cooperative Acute Stroke Study (including ECASS II, ECASS III, and author-defined criteria which were nearly the same as the ECASS II definition); NINDS, the National Institute of Neurological Disorders and Stroke study; SITS-MPST, the Safe Implementation of Thrombolysis in Stroke-Monitoring study. *References of studies that met the criteria of multicenter (>3 centers)^19,22–24^.

Eleven studies reported mortality within 3 months. None of them showed a significant difference between low dose and standard dose rt-PA. Heterogeneity across the studies was low (*P*_*hetero*_ = 0.83, I^2^ = 0), and no significant difference was found from a fixed-effects model (OR = 0.87, 95% CI 0.74–1.02, *P* = 0.08), as shown in Table 1 and Supplementary Figure 2.

**Figure 2:**
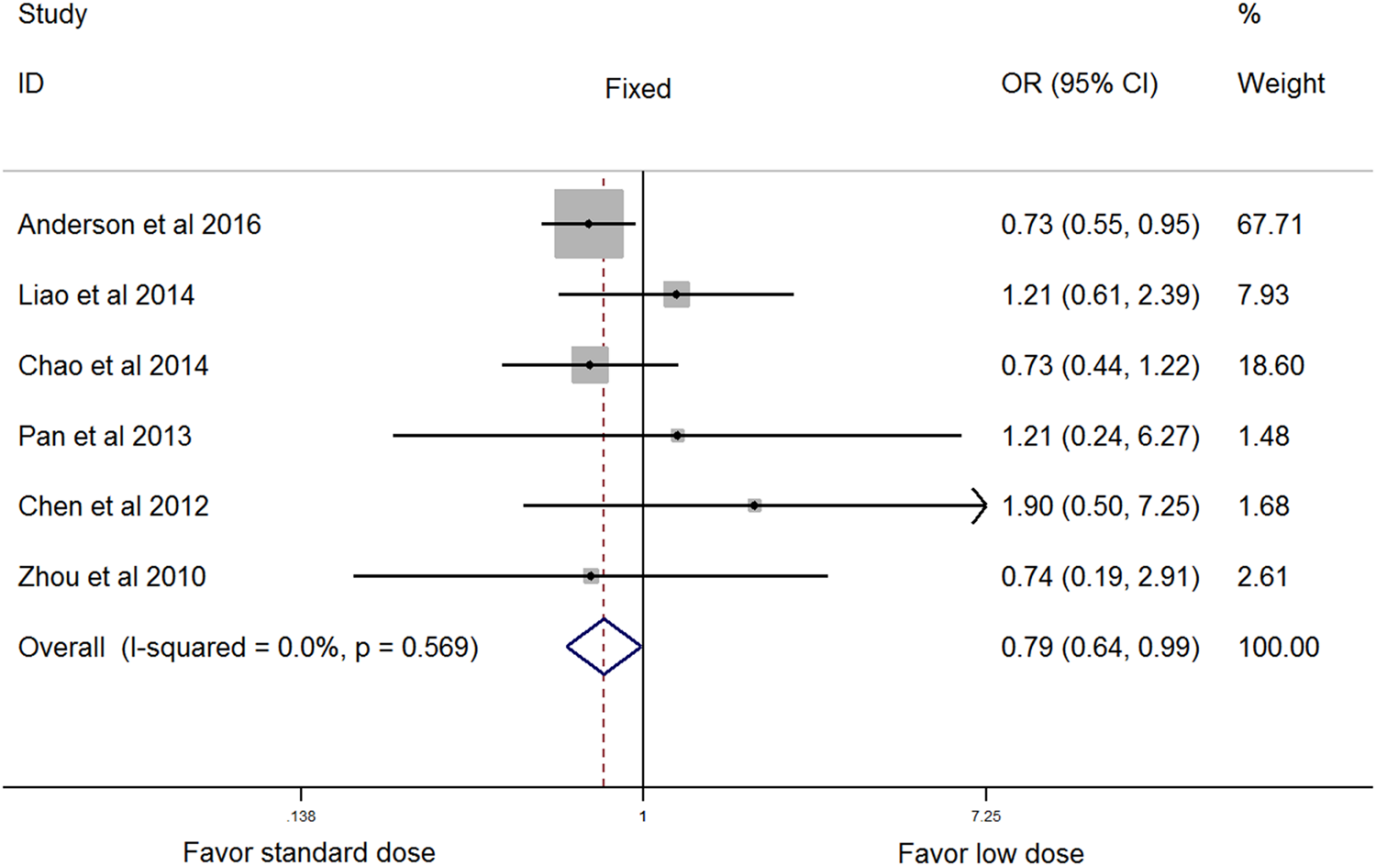
Forest plot showing pooled estimates for symptomatic intracerebral hemorrhage as defined by NINDS. NINDS, the National Institute of Neurological Disorders and Stroke study.

As shown in Supplementary Table 1, SICH was identified in 6 studies per ECASS definitions, with 4 per ECASS II definition^18,19,23,24^, and 2 per ECASS III definition^25,27^. SICH was also identified in 6 studies per NINDS definition^19,23–27^ and 4 studies per SITS-MOST definition^19,23,24,26^. In 3 other studies, SICH was identified per author-defined criteria^22,29,30^, which was nearly the same as the ECASS II definition. One study had zero cases of intracerebral hemorrhage occur^21^. Thus, pooled analyses for SICH were conducted separately based on ECASS (ECASS II, ECASS III, and author-defined criteria), SITS-MOST, and NINDS (Table 1, Fig. 2, and Supplementary Figures 3 and 4). When adopting the NINDS definition, no evidence of heterogeneity was found (*P*_*hetero*_ = 0.57, I^2^ = 0), and SICH was significantly lower in those receiving low dose rt-PA than in those receiving standard dose from both fixed- and random-effects models (both: OR = 0.79, 95% CI 0.64–0.99, *P* = 0.04).

### Subgroup Analysis

When only combining results from multicenter studies, the pooled estimate for favorable outcome had no heterogeneity (*P*_*hetero*_ = 0.61, I^2^ = 0), and was statistically significant from both fixed- and random-effects models (both: OR = 0.88, 95% CI 0.80–0.98, *P* = 0.02). For SICH defined by NINDS, there was no heterogeneity (*P*_*hetero*_ = 0.39, I^2^ = 0), and the pooled estimate was statistically significant using both fixed- and random-effects models (OR = 0.77, 95% CI 0.61–0.96, *P* = 0.02; OR = 0.77, 95% CI 0.61–0.97, *P* = 0.02, respectively). But no statistical significance was found for any outcome in non-multicenter studies (Table 1). When removing the non-Asian patients in Anderson’s study^19^ from meta-analysis, no significant difference was found between low dose and standard dose group in all outcomes (Table 2).

**Table 2:** Pooled estimates with non-Asian patients excluded.

Outcomes	NO. of Studies	NO. of Patients	NO. of low dose	NO. of standard dose	*P*_*hetero*_	I^2^	Fixed OR (95% CI)	Random OR (95% CI)
Favorable outcome	11	6076	1230/2842	1587/3594	0.01	57.2%	0.91 (0.82–1.01)	0.94 (0.77–1.15)
Mortality	11	6241	250/2951	370/3650	0.89	0	0.93 (0.78–1.11)	0.93 (0.78–1.12)
SICH by ECASS	9	6206	134/2713	180/3493	0.037	51.3%	1.01 (0.80–1.27)	0.93 (0.67–1.57)
SICH by NINDS	6	4416	123/2054	163/2362	0.51	0	0.79 (0.61–1.01)	0.79 (0.61–1.01)
SICH by SITS-MOST	4	4228	33/1936	43/2292	0.05	61.3%	0.90 (0.56–1.44)	1.12 (0.48–2.57)

ECASS, the European Cooperative Acute Stroke Study (including ECASS II, ECASS III, and author-defined criteria which were nearly the same as the ECASS II definition); NINDS, the National Institute of Neurological Disorders and Stroke study; SITS-MPST, the Safe Implementation of Thrombolysis in Stroke-Monitoring study. References of included studies^18,19*, 21– 27,29,30^. *Non-Asian patients in Anderson’s study (ref.^19^) were excluded.

### Sensitivity Analysis

The variation and range of the pooled ORs after removing a single study from the meta-analysis and repeating the process multiple times are listed in Supplementary Table 3.

The stability of the pooled estimate was assessed by removing some studies from the meta-analysis based on various exclusion criteria each time; this stability analysis is summarized in Supplementary Table 4. The pooled estimate for favorable outcome, mortality and SICH as defined by NINDS changed significantly depending on some of the exclusion criteria that was used.

### Publication Bias

**Figure 3:**
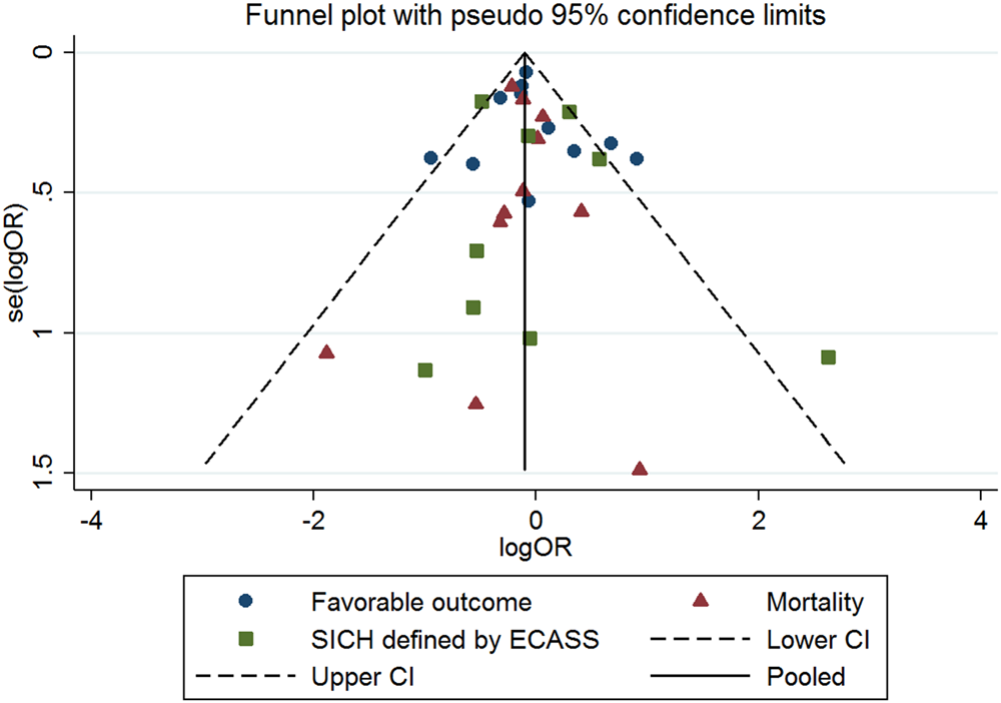
Funnel plot showing publication bias for favorable outcome, mortality and symptomatic intracerebral hemorrhage as defined by ECASS. ECASS, the European Cooperative Acute Stroke Study.

No substantial asymmetry was found in the funnel plot (Fig. 3). The Egger’s linear regression test indicated no publication bias for favorable outcome, mortality and SICH defined by ECASS (*P* = 0.592, 0.996 and 0.558, respectively). Publication bias for SICH as defined by NINIDS or SITS-MOST was not assessed, due to the small number of included studies (far less than 10).

## Discussion

We herein present a meta-analysis assessing the difference in IVT efficacy and safety between low dose and standard dose rt-PA, and found that low dose rt-PA may be related to fewer favorable outcomes at 3 months, and a lower incidence of SICH as defined by the NINDS. These results, however, lack stability.

A prior meta-analysis with 10 cohort studies involving 4,348 cases was conducted by Liu *et al*. in 2015 and, demonstrated that there was no significant difference in favorable outcome between low dose (<0.85 mg/kg) and standard dose (0.85–0.95 mg/kg) rt-PA using a random-effects model^32^. Two of the 10 cohort studies presented in Liu’s study were excluded from our meta-analysis: one for data overlapping entirely with another study of larger sample size^24,28^, and one for having only 2 patents receive low dose rt-PA^48^. The addition of 3 studies published recently^18,19,21^ allowed us to combine 11 studies involving 7,598 patients to evaluate the effect of rt-PA dose on favorable outcome. The pooled estimate showed that there was no significant difference in favorable outcome between low dose and standard dose rt-PA using a random-effects model, which was consistent with the prior meta-analysis.

Although only 4 studies met the criteria of multicenter research for favorable outcome, participants in these studies accounted for 84.7% (6438/7598) of all^19,22–24^. We therefore conducted a subgroup analysis that considered only multicenter studies, and found that standard dose rt-PA was significantly associated with an increased chance of favorable outcome using both fixed- and random-effects models. Additionally, sensitivity analysis demonstrated that pooled estimates for favorable outcome could reach a statistical significance according to some exclusion criteria (Supplementary Tables 3 and 4). Thus, it seemed that the association between standard dose rt-PA and increasing favorable outcome could not be denied by present results. But when interpreting these finding, we need to take into account that most of the included studies were conducted with nonrandomized design, which would cause a non-ignorable selection bias, where patients in low dose may have a fewer chance of favorable outcome. Since in clinical practices, patients at a higher risk of hemorrhage transformation on admission are more prone to be allocated to receiving lower dose rt-PA by the neurologists. And these patients usually have a larger infarct area or other adverse factors, which may decrease the chance of favorable outcome at 3 months. Therefore, the probable beneficial effect of standard dose on favorable outcome at 3 months need to be validated by more randomized controlled trials in future.

With respect to SICH, Liu’s meta-analysis found no significant difference in the incidence of SICH between low dose and standard dose rt-PA using a random-effects model^32^. Since there were many definitions of SICH among the various studies, which may challenge the reliability of pooled results, we in present meta-analysis combined results and conducted pooled estimates separately for SICH according to different definitions (ECASS, NINDS, and SITS-MOST). When using the definition from the NINDS, the incidence of SICH was found to be significantly higher in standard dose group than in low dose group using both fixed- and random-effects models. Among the included studies, only Anderson’s study involved parts of non-Asian patients^19^. When removing these non-Asian patients from meta-analysis, there was no significant difference between low dose and standard dose in incidence of SICH as defined by NINDS. Moreover, only Anderson’s study showed that incidence of SICH as defined by NINDS was significantly different between low dose (0.6 mg/kg) and standard dose (0.9 mg/kg) groups, with those in the standard dose group having a higher incidence of SICH^19^. Given that the sample size of Anderson’s study was much larger than the other studies, accounting for 58.5% (3,297/5,634) of all participants, the pooled estimate was undoubtedly dominated by this study. Thus, we also calculated the pooled estimate after removing Anderson’s study from the analysis despite the quality of this study being high with a NOS score of 9; removing it did not change the heterogeneity of the pooled analysis. And no significant difference in incidence of SICH as defined by NINDS was found after Anderson’s study removed (data not shown). In addition, when removing studies based on some exclusion criteria, the pooled estimate for SICH as defined by the NINDS failed to reach statistical significance (Supplementary Table 4). Thus the results regarding the association between rt-PA dose and SICH as defined by the NINDS need to be interpreted with caution, and further research is warranted to explore this topic.

The NINDS definition was thought to be overly inclusive, less precise, and of lowest inter-rater agreement in identifying SICH when compared to the ECASS II, ECASS III, and SITS-MOST definitions^51,52^. Even though the standard dose rt-PA may actually increase the incidence of SICH as defined by the NINDS, the implications of this on later outcomes at 3 months were inconclusive. Gumbinger’s study demonstrated that SICH as defined by the NINDS, as well as by the ECASS II, ECASS III, and SITS-MOST, was significantly associated with mortality at 3 months^52^. In Rao’s study, however, SICH as defined by the NINDS was not associated with the distribution of mRS scores or mortality at 3 months^51^. Furthermore, the prior meta-analysis^32^ and our present study found that mortality was not significantly different between low dose and standard dose rt-PA. These results suggest that low dose rt-PA may reduce the risk of SICH as defined by the NINDS, but may not decrease mortality at 3 months when compared to standard dose treatment. Given the possibility of the beneficial effects of standard dose treatment on favorable outcome at 3 months, a lower risk of SICH as defined by the NINDS may not alone be a sound reason to administer rt-PA at a low dose.

In developing Asian countries, in addition to worrying about the potentially higher risk of SICH compared to Western patients, financial burden on a family is another frequently-encountered barrier compelling the next-of-kin and neurologist to choose a low dose (i.e. one vial) of rt-PA. As Sila suggested, however, “using less effective therapies to save short-term costs will only increase the costs of long-term care for disabled stroke survivors”^53^. Therefore, until additional evidence is available, thrombolysis with low dose rt-PA for the purpose of being cost-conscious may not be an ideal choice.

Some limitations to our study must be acknowledged. Firstly, in some studies, low dose rt-PA administered because of a patient hitting the limit for maximum dose or inaccurate weight assessment can range widely, besides, parts of the participants in several studies may have been assigned to the wrong group based on a slightly different cut-off point than 0.85 mg/kg, of which both may obscure a true difference in outcomes between low dose and standard dose treatment. Future studies with pre-specified doses (i.e. 0.6 mg/kg versus 0.9 mg/kg) are warranted to avoid this limitation. Secondly, we conducted the pooled estimate for SICH by separately combining and analyzing studies based on different definitions of SICH (ECASS, NINDS, and SITS-MOST). This could decrease the clinical heterogeneity across studies, but also reduced the number of studies included in any given sub-analysis, which may weaken the reliability of the results. Thirdly, patients receiving endovascular recanalization treatment during thrombolysis was referred to in Kim’s study^22^, accounting for 26.5% of all patients, and was not clearly indicated in the other studies included in the meta-analysis. Given the disparity in medical and economic conditions and resources across countries and regions, the percentage of patients receiving both thrombolysis and endovascular recanalization treatment may differ substantially between studies, which may cause a non-negligible clinical heterogeneity. Fourthly, the nonrandomized design of most studies would cause a selection bias, then challenge the reliability of outcomes. But we did not conduct a subgroup analysis for randomized studies, due to the few number of studies. Finally, subgroup analysis by age, gender, baseline NIHSS score, time interval from stroke onset to IVT, or other clinical characteristics would possibly supply some additional meaningful results, but was not conducted in the present meta-analysis due to the diversity in the way the information was presented and reported across studies.

## Conclusion

The current limited published evidence was insufficient to support the speculation that low dose rt-PA was comparable to the standard dose in thrombolytic efficacy and safety in Asian stroke patients. However, results from the present meta-analysis lacked stability. Large-scale randomized controlled trials, or multicenter studies including a large sample of patients and pre-specified dosing are warranted in the future.

## Electronic supplementary material

Supplementary Figures and Tables
