# Supplementary material for: Efficacy and safety of low dose alteplase for intravenous thrombolysis in Asian stroke patients: a meta-analysis
Source: Sci Rep. 2017 Nov 22;7:16076. doi: 10.1038/s41598-017-16355-9 (PMC5700077; doi:10.1038/s41598-017-16355-9)
Supplement: Supplementary file 1 — Supplementary Figures and Tables [file 41598_2017_16355_MOESM1_ESM.pdf]

Efficacy and safety of low dose alteplase for intravenous thrombolysis in Asian stroke patients: a meta-analysis

Ge Tan, Haijiao Wang, Sihan Chen, Deng Chen, Lina Zhu, Da Xu, Yu Zhang & Ling Liu

Supplementary Figure 1. Forest plot showing pooled estimates for favorable outcome.

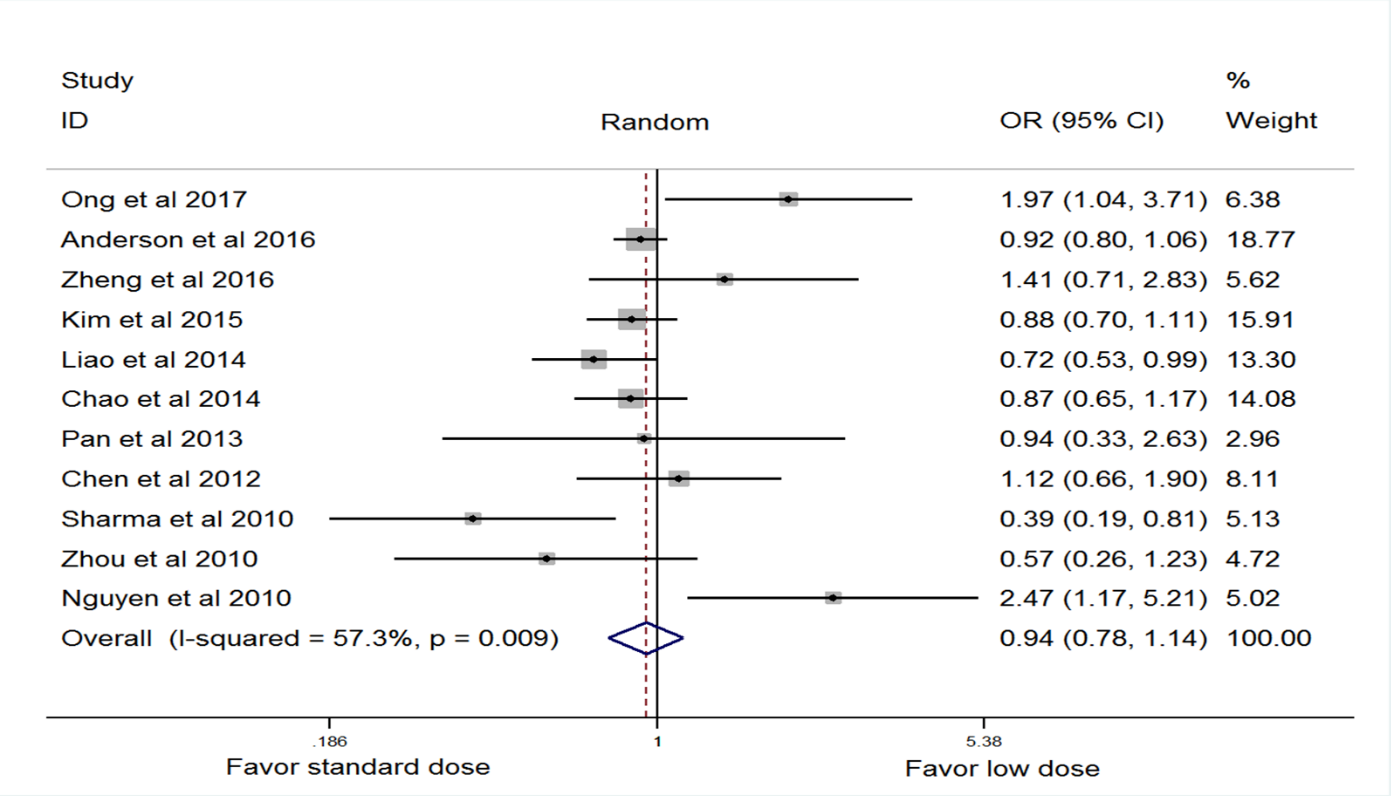

Supplementary Figure 2. Forest plot showing pooled estimates for mortality.

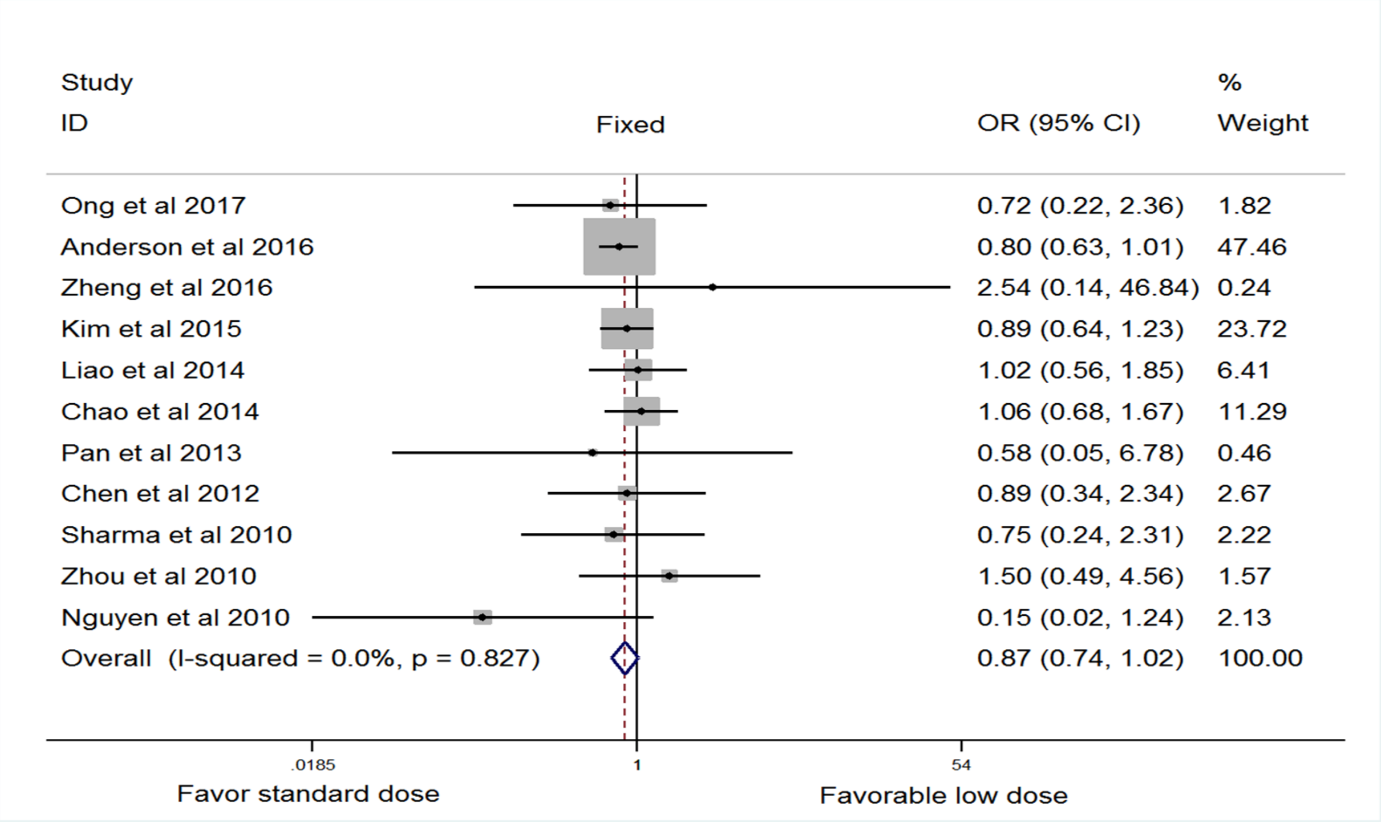

Supplementary Figure 3. Forest plot showing pooled estimates for symptomatic intracerebral hemorrhage as defined by ECASS.

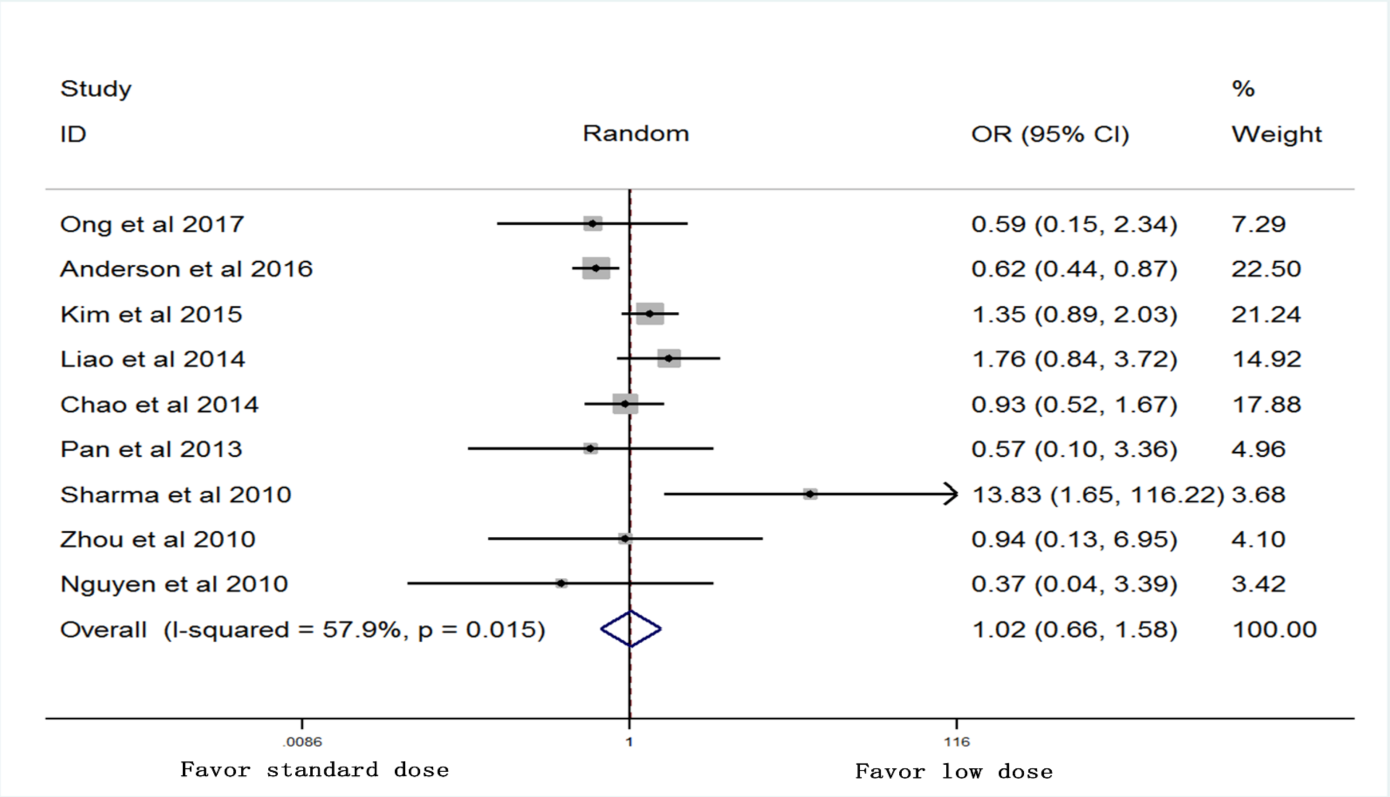

ECASS, the European Cooperative Acute Stroke Study (including ECASS II, ECASS III, and author-defined criteria which were nearly the same as the ECASS II definition).

Supplementary Figure 4. Forest plot showing pooled estimates for symptomatic intracerebral hemorrhage as defined by SITS-MOST.

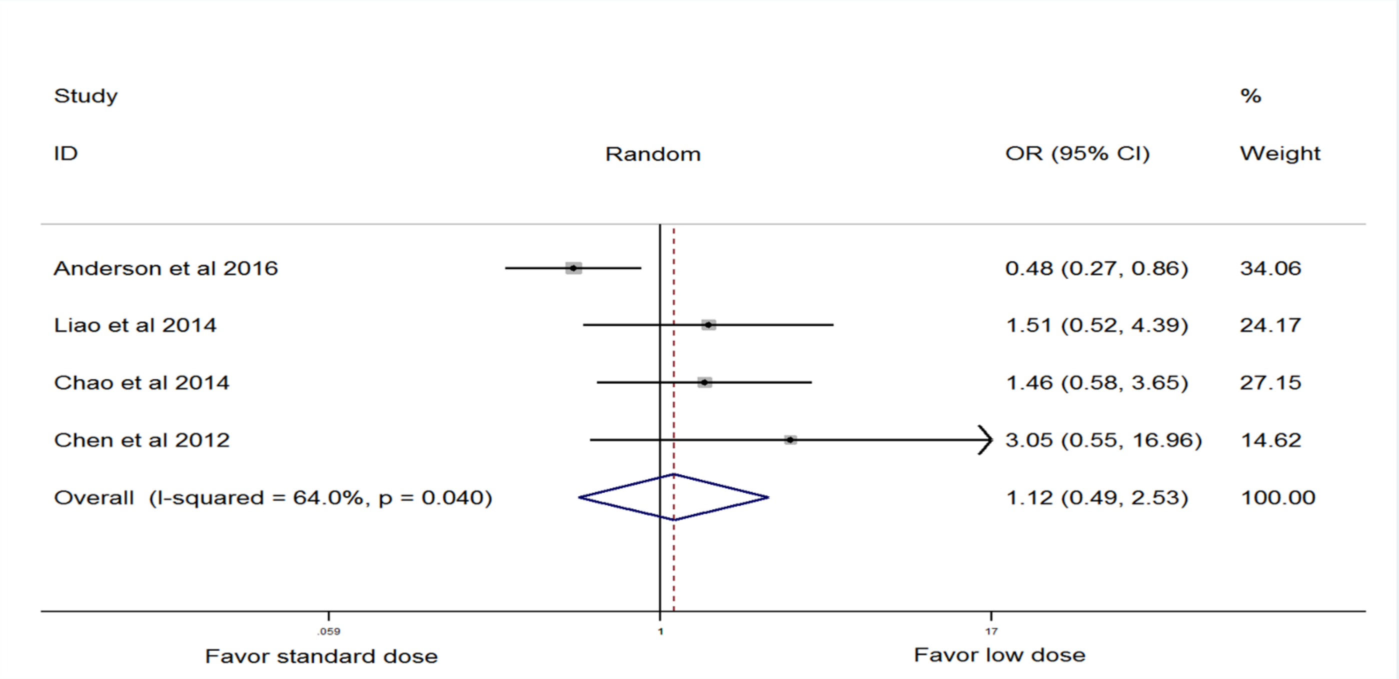

SITS-MOST, the Safe Implementation of Thrombolysis in Stroke-Monitoring study.

Supplementary Table 1. Characteristics of included cohort studies.

Ref	Study	Region	Centers, n	Design	Method of data collection	Female overall, %	Loss to follow-up at 3 months, %	rt-PA dose, mg/kg
**18**	Ong et al 2017	Taiwan China	1	Nonrandomized	Retrospectively	42.3	5.1	0.6
								0.7
								0.8
								0.9
**19**	Anderson et al 2016	International*	≈100	Randomized	Prospectively	37.9	2.8	0.6
								0.9
**21**	Zheng et al 2016	Mainland China	1	Randomized	Prospectively	36.8	0	0.6-0.7
								0.6-0.7
								0.9
**22**	Kim et al 2015	Korea	15	Nonrandomized	Prospectively	40.7	0	0.6
								0.9
**23**	Liao et al 2014	Mainland China	67	Nonrandomized	Prospectively	37.0	2.0	0.5-0.7
								0.7-0.85
								0.85-0.95
**24**	Chao et al 2014	Taiwan China	23	Nonrandomized	Prospectively	37.5	4.4	0.6
								0.7
								0.8
								0.9
**25**	Pan et al 2013	Mainland China	1	Nonrandomized	Retrospectively	39.8	0	<0.75
								0.75-0.90
								0.90
**26**	Chen et al 2012	Taiwan China	2	Nonrandomized	Prospectively	36.0	7.7	<0.85

								≥ 0.85
**27**	Zhou et al 2010	Mainland China	1	Nonrandomized	Retrospectively	48.6	0	0.6-0.7
								0.8
								0.9
**29**	Nguyen et al 2010	Vietnam	3	Nonrandomized	Prospectively	53.7	0	0.6–0.86
								0.9
**30**	Sharma et al 2010	Singapore	1	Nonrandomized	Prospectively	40.8	0	0.5–0.71
								0.9

Continue...

**Ref**	**Cases, n**	**Age at stroke onset, y †**	**Female, %**	**Baseline NIHSS scores †**	**Time interval from stroke onset to thrombolysis, Min †**	**Favorable outcome, n**	**Mortality, n**	**Symptomatic intracerebral hemorrhage, n ‡**
**18**	71	71.6±10.6	56.3	14.9±7.2	111.7±37.0	24/63	4/63	1 EII/71
	59	65.3±13.5	35.6	13.8±5.7	120.1±36.7	24/57	5/57	5 EII/59
	88	62.2±12.9	37.5	13.1±5.9	113.6±44.2	48/85	2/85	1 EII/88
	56	66.5±11.6	39.3	14.9±6.3	122.1±45.5	17/55	4/55	3 EII/56
**19**	1654	68(58-76)	38.3	8 (5-14)	170 (125-218)	752/1607	140/1607	55 EII, 17 S, 98 N/1654
	1643	67(58-76)	37.4	8 (5-14)	170 (127-219)	782/1599	170/1599	86 EII, 35 S, 131 N/1643
**21**	90	62.7±13.1	35.6	11.4 ± 4.3	195.8±62.3	65/90	2/90	0
	90	61.2±10.7	38.9	10.9±3.8	236.7±70.6	49/90	3/90	0
	40	63.5±12.2	35.0	11.5±5.5	240.3±68.1	22/40	0	0
**22**	450	69.0±12.7	45.6	13.9±7.0	126±54	146/450	57/450	38 Se/450
	1076	68.2±12.3	38.7	10.5±6.0	126±54	380/1076	151/1076	69 Se/1076
**23**	75	62 (52–71)	18.7	10 (7–17)	175.2 (151.8–190.8)	31/74	14/74	0 EII, 0 N, 0 S/75
	131	68 (57–73)	43.5	10 (6–15)	175.8 (139.8–199.8)	61/127	11/127	11 EII, 12 N, 5 S/131

	678	63 (55–72)	37.8	11 (7–15)	167.4 (139.8–195.0)	258/665	49/666	21 EII, 33 N, 11 S/678
**24**	181	70.1±10.9	42.0	14.4±6.1	147.7±41.7	56/146	14/181	10 EII, 10 N, 5 S/181
	199	70.1±10.9	38.2	14.7±6.4	140.4±36.7	44/156	19/199	6 EII, 10 N, 4 S/199
	202	66.9±13.5	36.6	15.2±6.5	145.5±36.0	46/171	18/202	11 EII, 12 N, 5S/202
	422	66.1±12.0	35.6	15.0±7.7	137.3±57.3	124/367	35/422	21 EII, 31 N, 7S/422
**25**	31	63.8±9.3	24.2	8.7±4.6	185.3±54.4	16/31	1/31	1 EIII, 3N/31
	33	64.5±7.7	45.2	9.2±5.0	190.4±62.2	20/33	1/33	3 EIII, 5N/33
	19	65.7±9.3	52.7	11.1±5.8	177.2±44.0	11/19	1/19	2 EIII, 2N/19
**26**	105	67.9±12.8	38.1	13.3±6.2	144±41	39/95	7/95	5N, 4S/105
	156	67.9±12.3	34.6	13.1±6.3	141±39	56/146	12/146	4N, 2S/156
**27**	23	69.8±8.6	13.0	12.6±6.8	170.3±43.9	8/23	4/23	1 EIII, 1 N/23
	31	72.9±8.7	51.6	12.7±5.0	174.3±45.2	12/31	5/31	1 EIII, 3 N/31
	51	72.7±10.7	62.7	13.0±6.3	153.5±53.0	26/51	6/51	2 EIII, 5 N/51
**29**	48	57 ± 13	69.0	10.5 (5.75)	141±33	27/48	1/48	1 Se/48
	73	58 ± 14	44.0	12 (7)	145±33	25/73	9/73	4 Se/73
**30**	48	55 ± 12	46.0	12 (10)	165 (30)	17/48	5/48	7 Se/48
	82	62 ± 13	38.0	15 (11)	155 (47)	48/82	11/82	1 Se/82

Ref, reference; rt-PA, recombinant tissue plasminogen activator; NIHSS, National Institute of Health stroke scale.

* Including 63.2% patients from China and other Asia countries, and 36.6% patients from Europe, Austrilia, and South America. Data of outcomes for Asian patients in this study: favorable outcome (low dose: 497/1024 vs. standard dose: 520/1020), Mortality (87/1024 vs. 92/1020), ECASS II (37/1043 vs. 57/1036), NINDS (62/1043 vs. 88/1036), SITS-MOST (10/1033 vs. 23/1036).

† Data were shown as mean ± SD or median with interquartile range.

‡ EII, EIII, N, S and Se meant identifying symptomatic intracerebral hemorrhage per the European Cooperative Acute Stroke Study (ECASS II), ECASS III, the National Institute of Neurological Disorders and Stroke study (NINDS), the Safe Implementation of Thrombolysis in Stroke-Monitoring study (SITS-MOST) and author-defined criteria, respectively.

Supplementary Table 2. Quality assessment of included cohort studies with Newcastle-Ottawa Scale.

Ref	Selection				Comparability	Outcome			Total scores
	Representativeness of the Exposed Cohort	Selection of the Non-Exposed Cohort	Ascertainment of Exposure	Outcome of Interest Not Present at Start of Study	Design or Analysis	Assessment of Outcome	Length of Follow-Up	Adequacy of Follow-Up	
**18**	1	1	1	1	1	1	1	1	8
**19**	1	1	1	1	2	1	1	1	9
**21**	1	1	1	1	1	1	1	1	8
**22**	1	1	1	1	1	1	1	1	8
**23**	1	1	1	1	1	1	1	1	8
**24**	1	1	1	1	0	1	1	1	7
**25**	1	1	1	1	1	1	1	1	8
**26**	1	0	1	1	1	1	1	0	6
**27**	1	1	1	1	1	1	1	1	8
**29**	1	1	1	1	1	1	1	1	8
**30**	1	0	1	1	1	1	1	1	7

Ref, reference.

Supplementary Table 3. Variation range of pooled ORs when removing a single study from meta-analysis each time and corresponding studies.

Model	Outcome measures	Before removing	After removing			
			Minimum		Maximum	
		OR (95% CI)	Study removed	OR (95% CI)	Study removed	OR (95% CI)
Fixed	Favorable outcome	0.91 (0.83 – 1.00)	Ong et al 2017	**0.89 (0.81 – 0.98)**	Liao et al 2014	0.93 (0.84 – 1.03)
	Mortality	0.87 (0.74 – 1.02)	Chao et al 2014	0.84 (0.71 – 1.00)	Anderson et al 2016	0.92 (0.74 – 1.15)
	SICH by ECASS	0.93 (0.75 – 1.16)	Kim et al 2015	0.81 (0.63 – 1.05)	Anderson et al 2016	1.26 (0.94 – 1.67)
	SICH by NINDS	**0.79 (0.64 – 0.99)**	Liao et al 2014	**0.76 (0.60 – 0.95)**	Anderson et al 2016	0.93 (0.65 – 1.35)
	SICH by SITS-MOST	0.82 (0.54 – 1.25)	Chao et al 2014	0.70 (0.43 – 1.13)	Anderson et al 2016	1.65 (0.87 – 3.13)
Random	Favorable outcome	0.94 (0.78-1.14)	Nguyen et al 2010	0.89 (0.76 – 1.06)	Liao et al 2014	0.98 (0.80 – 1.22)
	Mortality	0.87 (0.74 – 1.02)	Chao et al 2014	0.85 (0.71 – 1.00)	Anderson et al 2016	0.93 (0.75 – 1.16)
	SICH by ECASS	1.02 (0.66 – 1.58)	Liao et al 2014	0.92 (0.58 – 1.48)	Anderson et al 2016	1.19 (0.79 – 1.81)
	SICH by NINDS	**0.79 (0.64 – 0.99)**	Liao et al 2014	**0.76 (0.60 – 0.95)**	Anderson et al 2016	0.93 (0.65 – 1.35)
	SICH by SITS-MOST	1.12 (0.49 – 2.53)	Chan et al 2012	0.94 (0.40 – 2.17)	Anderson et al 2016	1.64 (0.86 – 3.12)

ECASS, the European Cooperative Acute Stroke Study (including ECASS II, ECASS III, and author-defined criteria which were nearly the same as the ECASS II definition); NINDS, the National Institute of Neurological Disorders and Stroke study; SITS-MPST, the Safe Implementation of Thrombolysis in Stroke-Monitoring study.

Supplementary Table 4. Stability assessment of pooled estimates by removing some studies.

Excluding Criteria	Outcomes	NO. of Studies	NO. of Patients	NO. of low dose	NO. of Standard dose	P_{hetero}	I^2	Fixed OR (95%CI)	Random OR (95%CI)
Overall	Favorable outcome	11	7598	1485/3425	1849/4173	0.01	57.3%	0.91 (0.83 – 1.00)	0.94 (0.78 – 1.14)
	Mortality	11	7763	303/3534	448/4229	0.83	0	0.87 (0.74 – 1.02)	0.87 (0.74 – 1.02)
	ECASS	9	7424	152/3324	210/4100	0.02	57.9%	0.93 (0.75 – 1.16)	1.02 (0.66 – 1.58)
	NINDS	6	5634	159/2665	206/2969	0.57	0	**0.79 (0.64 – 0.99)**	**0.79 (0.64 – 0.99)**
	SITS-MOST	4	5446	40/2547	55/2899	0.04	64.0%	0.82 (0.54 – 1.25)	1.12 (0.49 – 2.53)
Criteria Ⅰ	Favorable outcome	8	6368	1226/2699	1660/3669	0.08	44.1%	0.91 (0.82 – 1.01)	0.94 (0.78 – 1.13)
	Mortality	8	6369	236/2699	398/3670	0.67	0	0.85 (0.71 – 1.01)	0.85 (0.71 – 1.01)
	ECASS	6	6018	111/2478	185/3540	0.03	58.4%	0.89 (0.69 – 1.13)	0.97 (0.58 – 1.61)
	NINDS	5	4630	127/2083	175/2547	0.44	0	0.81 (0.63 – 1.03)	0.81 (0.64 – 1.03)
	SITS-MOST	3	4442	26/1965	48/2477	0.04	69.1%	0.70 (0.43 – 1.13)	1.07 (0.36 – 3.19)
Criteria Ⅱ	Favorable outcome	5	4897	1164/2608	1049/2289	0.03	61.0%	0.94 (0.83 – 1.05)	0.98 (0.75 – 1.29)
	Mortality	5	5061	219/2717	232/2344	0.87	0	0.85 (0.70 – 1.04)	0.85 (0.70 – 1.04)
	ECASS	4	4705	96/2502	112/2203	0.03	67.1%	0.75 (0.57 – 1.00)	0.93 (0.46 – 1.88)
	NINDS	3	4562	135/2341	166/2221	0.39	0	**0.75 (0.59 – 0.95)**	**0.75 (0.59 – 0.95)**
	SITS-MOST	3	4562	35/2341	44/2221	0.03	71.1%	0.74 (0.47 – 1.17)	1.06 (0.37 – 3.03)
Criteria Ⅲ	Favorable outcome	9	6538	1225/2772	1703/3766	0.01	63.3%	0.91 (0.82 – 1.01)	0.93 (0.74 – 1.18)
	Mortality	9	6539	247/2772	413/3767	0.81	0	**0.84 (0.70 – 0.99)**	0.84 (0.71 – 1.00)
	ECASS	8	6420	125/2742	189/3678	0.01	63.2%	0.93 (0.74 – 1.18)	1.05 (0.61 – 1.80)
	NINDS	5	4630	127/2083	175/2547	0.44	0	0.81 (0.63 – 1.03)	0.81 (0.64 – 1.03)
	SITS-MOST	3	4442	26/1965	48/2477	0.04	69.1%	0.70 (0.43 – 1.13)	1.07 (0.36 – 3.19)
Criteria Ⅳ	Favorable outcome	9	7392	1438/3323	1798/4049	0.06	47.1%	0.90 (0.82 – 1.00)	0.91 (0.77 – 1.08)
	Mortality	9	7537	292/3432	433/4105	0.97	0	0.87 (0.74 – 1.02)	0.87 (0.74 – 1.02)

	ECASS	7	7198	149/3222	204/3976	0.01	67.3%	0.94 (0.76 – 1.18)	1.07 (0.66 – 1.73)
	NINDS	5	5529	155/2611	201/2918	0.43	0	**0.80 (0.64 – 0.99)**	**0.80 (0.64 – 0.99)**
	SITS-MOST	NC	-	-	-	-	-	-	-
Criteria Ⅴ	Favorable outcome	10	7515	1449/3361	1838/4154	0.01	61.6%	0.91 (0.83 – 1.01)	0.95 (0.77 – 1.16)
	Mortality	10	7680	301/3470	447/4210	0.76	0	0.87 (0.74 – 1.02)	0.87 (0.74 – 1.02)
	ECASS	8	7341	148/3260	208/4081	0.01	62.6%	0.94 (0.75 – 1.17)	1.05 (0.66 – 1.68)
	NINDS	5	5551	151/2601	204/2950	0.46	0	**0.79 (0.63 – 0.98)**	**0.79 (0.63 – 0.98)**
	SITS-MOST	NC	-	-	-	-	-	-	-
Criteria Ⅵ	Favorable outcome	10	7378	1371/3245	1827/4133	0.01	58.8%	0.90 (0.82 – 1.00)	0.92 (0.76 – 1.12)
	Mortality	10	7543	298/3354	448/4189	0.80	0	0.86 (0.73 – 1.01)	0.86 (0.73 – 1.01)
	ECASS	NC	-	-	-	-	-	-	-
	NINDS	NC	-	-	-	-	-	-	-
	SITS-MOST	NC	-	-	-	-	-	-	-
Criteria Ⅶ	Favorable outcome	9	7097	1350/3125	1776/3972	0.03	52.7%	**0.89 (0.80 – 0.98)**	0.88 (0.73 – 1.06)
	Mortality	9	7262	285/3234	432/4028	0.67	0	0.87 (0.74 – 1.02)	0.87 (0.74 – 1.03)
	ECASS	NC	-	-	-	-	-	-	-
	NINDS	NC	-	-	-	-	-	-	-
	SITS-MOST	NC	-	-	-	-	-	-	-
Criteria VIII	Favorable outcome	8	7150	1333/3102	1795/4048	0.02	56.9%	**0.90 (0.82 – 0.99)**	0.91 (0.76 – 1.11)
	Mortality	8	7315	281/3211	437/4104	0.69	0	0.86 (0.73 – 1.01)	0.86 (0.73 – 1.02)
	ECASS	6	6962	139/2988	203/3974	0.003	72.6%	0.95 (0.76 – 1.19)	1.13 (0.66 – 1.93)
	NINDS	4	5446	147/2547	199/2899	0.31	16.6%	**0.79 (0.63 – 0.99)**	0.82 (0.62 – 1.08)
	SITS-MOST	NC	-	-	-	-	-	-	-
Criteria IX	Favorable outcome	9	7394	1422/3313	1813/4081	0.04	51.4%	**0.90 (0.81 – 0.99)**	0.89 (0.75 – 1.07)
	Mortality	9	7559	300/3422	438/4137	0.93	0	0.88 (0.75 – 1.04)	0.88 (0.75 – 1.03)
	ECASS	7	7220	147/3212	204/4008	0.01	66.7%	0.95 (0.76 – 1.19)	1.10 (0.68 – 1.78)

NINDS	5	5551	151/2601	204/2950	0.46	0	**0.79 (0.63 – 0.98)**	**0.79 (0.63 – 0.98)**
SITS-MOST	NC	-	-	-	-	-	-	-

NC, meaning no change of the numbers of included studies.

ECASS, the European Cooperative Acute Stroke Study (including ECASS II, ECASS III, and author-defined criteria which were nearly the same as the ECASS II definition);

NINDS, the National Institute of Neurological Disorders and Stroke study; SITS-MPST, the Safe Implementation of Thrombolysis in Stroke-Monitoring study.

Overall, including **18, 19, 21, 22, 23, 24, 25, 26, 27, 29, 30**;

Criteria I, no difference in age across groups, including **19, 21, 22, 23, 25, 26, 27, 29**;

Criteria II, no difference in gender across group, including **18, 19, 21, 24, 26, 30**;

Criteria III, no difference in interval from stroke onset to thrombolysis, including **18, 19, 22, 23, 25, 26, 27, 29, 30**;

Criteria IV, overall percentage of female < 45%, including **18, 19, 21, 22, 23, 24, 25, 26, 30**;

Criteria V, sample size > 100, including **18, 19, 21, 22, 23, 24, 26, 27, 29, 30**;

Criteria VI, time interval from stroke onset to thrombolysis < 4.5 h, including **18, 19, 22, 23, 24, 25, 26, 27, 29, 30**;

Criteria VII, percentage of loss to follow-up < 5% at 3 months, including **19, 21, 22, 23, 24, 25, 27, 29, 30** for favorable outcome and Mortality, and additional **18, 26** for symptomatic intracerebral hemorrhage;

Criteria VIII, data collected prospectively, including **19, 21, 22, 23, 24, 26, 29, 30**;

Criteria IX, no cases being assigned to wrong group according to the cut-off point of 0.85 km/kg, including **18, 19, 21, 22, 23, 24, 26, 27, 30**.

Supplementary Table 5. Brief description of included studies.

Ref	Study	Brief description
**18**	Ong et al 2017	This was a single-center study of nonrandomized design in Taiwan China to assess the outcome of IVT with different rt-PA dose (0.6, 0.7, 0.8 or 0.9 mg/kg) and other clinical factors on IVT outcomes. A total of 274 patients with 42.3% females were collected retrospectively. Age and stroke subtypes were significantly different across groups. Data of SICH (ECASS II definition) after thrombolysis, and distribution of mRS scores and good outcome (mRS≤2) at discharge and 6 months were supplied. Information about favorable outcome (mRS 0-1), mortality and rate of loss to follow-up (5.1%) at 3 months were obtained after contacting with the author.
**19**	Anderson et al 2016	This was an international multi-center (≈100 centers) study of randomized design to assess the impact of different dose rt-PA (0.6 vs. 0.9 mg/kg) on IVT outcomes. A total of 3310 patients were registered prospectively, but only 3297 patients (63.2% Asian patients) with 37.9% females were included into analysis. There were no significantly different in baseline characteristics between groups. The study supplied data of many outcome measures including SICH (ECASS II, ECASS III, NINDS, SITS-MOST) after thrombolysis, and distribution of mRS scores, death/disability (mRS 2-6), death/major disability (mRS 3-6) and death at 3 months and so on. Data of outcomes just for Asian or non-Asian patients were also supplied in supplementary files except for favorable outcome (mRS 0-1), which were obtained after contacting with the author. Rate of loss to follow-up was 2.8% by calculating.
**21**	Zheng et al 2016	This was a single-center study of randomized design in Mainland China to assess the efficacy and safety of hypernormal shortened door to needle time plus different rt-PA dose (0.6-0.7 versus 0.9 mg/kg) therapy. A total of 220 patients with 36.8% females were collected prospectively. Period of receiving rt-PA and administration of antiplatelet drug were significantly different between groups. No cerebral hemorrhage occurred after thrombolysis. Data of good prognosis (mRS 0-1) and death at 3 months were supplied. Rate of loss to follow-up was identified to be zero by reviewing the study.
**22**	Kim et al 2015	This was a multi-center (15 centers) study of nonrandomized design in Korea to assess the efficacy and safety of IVT at different rt-PA dose (0.6 vs. 0.9 mg/kg). A total of 1526 patients with 40.7% females were collected prospectively. Gender, onset-to arrival delay, NIHSS score at arrival, stroke subtypes, location of arterial occlusion, endovascular recanalization treatment, atrial fibrillation, prestroke antiplatelets, prestroke anticoagulants, total cholesterol and diastolic blood pressure were significantly different between groups. Data of SICH (author-defined criteria) after thrombolysis, and distribution of mRS scores, mRS scores 0-1 and mortality at 3 months were supplied. Rate of loss to follow-up was identified to be zero by reviewing the study.
**23**	Liao et al	This was a multi-center (67 centers) study of nonrandomized design in Mainland China to assess whether IVT at standard dose was better than

	2014	that at low dose. A total of 919 patients were enrolled prospectively, and administered with different rt-PA dose (<0.5, 0.5-0.7, 0.7-0.85, 0.85-0.95 and ≥0.95 mg/kg). But only 884 patients (excluding patients receiving rt-PA of <0.5 or ≥0.95 mg/kg) with 37.0% females were included into analysis. Gender, independence (mRS scores 0-1) before stroke onset and hyperlipidemia were significantly different across groups. Data including SICH (ECASS II, NINDS and SITS-MOST), mortality at 7 d, and distribution of mRS scores, mRS scores 0-1, mRS scores 0-2 and mortality at 3 months were supplied. Rate of loss to follow-up was 2.0% by calculating.
**24**	Chao et al 2014	This was a multi-center (23 centers) study of nonrandomized design in Taiwan China to assess the effect of IVT at different rt-PA dose (0.6, 0.7, 0.8 or 0.9 mg/kg) on outcomes. A total of 1004 patients with 37.5% females were collected prospectively. Age, hyperlipidemia, atrial fibrillation, smoking and time to treatment were significantly different across groups. Data of SICH (ECASS II, NINDS, SITS-MOST) after thrombolysis, and mRS scores 0-1, mRS scores 0-2, mRS scores 5-6 at 3 months were supplied. Percentage of patients completing the 3-monghs follow-up were reported to be 95.6%, then the rate of loss to follow-up was 4.4%.
**25**	Pan et al 2013	This was a single-center study of nonrandomized design in Mainland China to assess the efficacy and safety of IVT with modified rt-PA regimen (<0.75, 0.75-0.90, 0.90 mg/kg). A total of 83 patients with 39.8% females were registered retrospectively. Only several baseline characteristics were included, and gender was significantly different across groups. Data of SICH (ECAIII, NINDS) after thrombolysis, and distribution of mRS scores and mortality at 3 months were supplied. Rate of loss to follow-up was identified to be zero by reviewing the study.
**26**	Chen et al 2012	This was a double-center study of nonrandomized design in Taiwan China to explore the optimal rt-PA dose (<0.85 vs. ≥ 0.85 mg/kg). A total of 261 patients with 36.0% females were collected prospectively. Emergency room to rt-PA and stroke subtypes were significantly different between groups. Data of SICH (NINDS, SITS-MOST, parenchymal hemorrhage 1 and parenchymal hemorrhage 2) after thrombolysis, and in-hospital mortality, mRS scores 0-2 at discharge, and distribution of mRS scores at 3 months were supplied. Rate of loss to follow-up was 7.7% by calculating.
**27**	Zhou et al 2010	This was a single-center study of nonrandomized design in Mainland China to assess the efficacy and safety of IVT with different rt-PA dose (0.6-0.7, 0.8 and 0.9 mg/kg). A Total of 105 patients with 48.6% females were included retrospectively. A few of baseline characteristics were collected, and gender and systolic pressure were significantly different across groups. Data of SICH (ECASS III and NINDS) after thrombolysis, and distribution of mRS scores, mRS scores 0-1 and mortality at 3 months were supplied. Rate of loss to follow-up was identified to be zero by reviewing the study.
**29**	Nguyen et al 2010	This was a three-center study of nonrandomized design in Vietnam to investigate the outcome of IVT with different rt-PA dose (0.6–0.86 and 0.9 mg/kg). A total of 121 patients with 53.7% females were collected prospectively. Among baseline characteristics only gender was significantly

		different between groups. Data of SICH (author-defined criteria) after thrombolysis, and mRS scores 0-1 and mortality at 3 months were supplied. Rate of loss to follow-up was identified to be zero by reviewing the study.
**30**	Sharma et al 2010	This was a single-center study of nonrandomized design in Singapore to assess the feasibility and safety of IVT in multiethnic Asian. A total of 130 patients with 40.8% females were collected prospectively, and assigned to receive different rt-PA dose (0.5–0.71 and 0.9 mg/kg). Age, hypertension, hypercholesterolemia, atrial fibrillation, NIHSS score, pretreatment systolic and diastolic blood pressure and stroke subtypes were significantly different between groups. Data of SICH (author-defined criteria) after thrombolysis, and functional independence (mRS scores 0-1) and mortality at 3 months were supplied. None of patients included was lost to follow-up.

Ref, reference; IVT, intravenous thrombolysis; rt-PA, recombinant tissue plasminogen activator; SICH, symptomatic intracerebral hemorrhage; NIHSS, National Institute of Health stroke scale; mRS, modified Rankin Scale; ECASS, the European Cooperative Acute Stroke Study; NINDS, the National Institute of Neurological Disorders and Stroke study; SITS-MOST, the Safe Implementation of Thrombolysis in Stroke-Monitoring study.
